# Beta-adrenergic receptor stimulation, histamine receptor inhibition, and potassium channel opening contribute to the relaxant effects of crocetin on airway smooth muscle

**DOI:** 10.22038/ijbms.2024.77720.16822

**Published:** 2024

**Authors:** Sepideh Behrouz, Arghavan Memarzia, Mohammad Hossein Eshaghi Ghalibaf, Mohammad Hossein Boskabady

**Affiliations:** 1 Applied Biomedical Research Center, Mashhad University of Medical Sciences, Mashhad, Iran; 2 Department of Physiology, Faculty of Medicine, Mashhad University of Medical Sciences, Mashhad, Iran; 3 Saffron Institute, University of Torbat Heydariyeh, Torbat Heydariyeh, Iran

**Keywords:** Airway smooth muscle, Crocetin, Cyclooxygenase, Histamine receptors, Potassium channels, Relaxant effects

## Abstract

**Objective(s):**

In the present study, the relaxant effect of crocetin on tracheal smooth muscle cells (TSM) and its possible mechanisms were evaluated.

**Materials and Methods:**

The study was conducted on 54 male Wistar rats in 8 groups. TSM was contracted by methacholine (10 μM) and KCl (60 mM), and the relaxant effects of four cumulative concentrations of crocetin, petal extract of saffron, and theophylline were examined on non-incubated and TSM incubated with propranolol, chlorpheniramine, diltiazem, atropine, glibenclamide, and indomethacin were investigated.

**Results:**

In non-incubated TSM contracted by methacholine or KCl, crocetin and theophylline showed concentration-dependent relaxant effects (all, *P*<0.001). However, various concentrations of crocetin showed significantly lower relaxant effects compared to those of theophylline (all, *P*<0.001). In the methacholine-induced contraction of TSM, the relaxation effect of the last concentration of crocetin in the TSM incubated with propranolol was lower than in non-incubated TSM (*P*<0.05). In the incubated TSM with chlorpheniramine, the relaxant effects of the two last concentrations of crocetin were significantly lower than in the non-incubated tissues contracted by KCl (*P*<0.05 and *P*<0.0). The levels of EC50 crocetin in the incubated TSM with glibenclamide, chlorpheniramine, and indomethacin were markedly lower than in non-incubated (all, *P*<0.05).

**Conclusion:**

The results showed potent relaxation effects of crocetin on TSM and were suggested to be through stimulation of ß-adrenergic receptors, inhibition of histamine (H_1_) receptors, and potassium channel opening mechanisms.

## Introduction

Bronchial asthma is an airway inflammatory disease that affects millions of people all over the world characterized by intermittent chronic inflammation, airway remodeling, and bronchospasms (1, 2). Airway hyper-responsiveness (AHR) is known as one of the main characteristics of asthma which is associated with lung dysfunction and is determined by the excessive contractile response of airway smooth muscle (ASM) to relatively little provocation which can lead to bronchoconstriction and airflow obstruction (3, 4). AHR may also be related to a lower release of relaxant agents such as vasoactive intestinal peptide (VIP) and adrenaline (5, 6). Therefore, prescribing bronchodilator drugs such as β2 agonists to relax bronchoconstriction in combination with anti-inflammatory drugs such as corticosteroids is known as the first line of asthma treatment (7). However, the systemic side effects of these drugs have prompted researchers to use available plant sources for the treatment of asthma (8).

Medicinal plants with properties such as anti-inflammatory, immunomodulatory, antihistaminic, and smooth-muscle relaxants can be considered as alternative treatments for asthma (9). Saffron) *Crocus sativus* L, *C. sativus*), is one of the most well-known medicinal plants used in Iranian traditional medicine for the treatment of various diseases such as asthma (10). In Avicenna’s most important medical book (Canon of Medicine) the effects of saffron on lung diseases, including asthma, are mentioned (11). Crocetin (C_2_0H_24_O_4_) is the major bioactive component of saffron, which has been recognized as a potent antioxidant (12)**,** anti-inflammatory (13), and anti-cancer (14). Crocetin has been reported to reduce the severity of asthma in animal models by modulating the activity of regulatory T (Treg) cells (15). Crocetin treatment also inhibited lung carcinogenesis induced by benzo (a) pyrene (16), radiation-induced lung injury (17), and lipopolysaccharide-induced acute lung injury (18) in experimental studies. Additionally, the pro-relaxing action of crocetin on aortic smooth muscle cells has been proven in the experimental model of hypertension (19).

Nevertheless, despite the many studies carried out on the biological effects of saffron ingredients, there is no scientific data about the relaxant effect of crocetin on airway smooth muscle. The present study was undertaken to evaluate the relaxant property of crocetin on rat tracheal smooth muscle (TSM) and its possible mechanisms.

## Materials and Methods

***Animals group***

Sixty-four male Wistar rats (200±20 g) were kept at 22±2 °C, the humidity at 50 to 60%, 12 hr of light, and 12 hr of dark in the Animal Breeding Center of Mashhad University of Medical Sciences. The experiments were approved by the Ethics Committee of Mashhad University of Medical Sciences (970790). The study was randomly performed in the following groups:

A) Non-incubated and incubated TSM contracted with methacholine (10 μM) including;

1) Non-incubated tissues (n=6 for crocetin and theophylline and n=5 for petal extract of saffron).

2) Incubated tissue with diltiazem (5 μM) (n=7), to investigate the calcium channel inhibitory effect.

3) Incubated tissue with glibenclamide (1 μM) (n=7), to investigate the potassium channel inhibitory effect.

4) Incubated tissue with propranolol (1 μM) (n=7), to investigate the effect of inhibiting beta-adrenergic receptors.

B) Non-incubated and incubated TSM contracted with KCl (60 mM), (20) including; 

5) Non-incubated tissues (n=6 for crocetin and theophylline and n=5 for petal extract of saffron).

6) Incubated tissue with atropine (1 μM), (n=7), to investigate the effect of cholinergic receptor inhibitory effect.

7) Incubated tissue with indomethacin (1 μM), (n=7), to investigate the cyclooxygenase inhibitory effect.

8) Incubated tissue with chlorphenamine (1 μM) (n=7), to investigate the histamine receptors inhibitory effect.

The relaxant effects of theophylline and petal extract of saffron were examined only in two non-incubated groups.

***Tissue preparation***

After anesthetizing rats by intraperitoneal (IP) administration of 50 ml/kg ketamine, the rats were sacrificed and their chests were opened. According to a previous study, the tissue of the trachea was prepared and the relaxant effect of crocetin was investigated (20).

***Preparation of the crocetin***

Concentrations of the crocetin (0.02, 0.06, 0.14, and 0.3 mg/ml), purchased from a market in Mashhad, Iran, were prepared by dissolving in saline.

***Preparation of the petal extract of saffron***

Aqueous-alcoholic extract of the petal of saffron was prepared and concentrated as previously described (21). Different concentrations of the extract (0.2, 0.4, 0.8, and 1 mg/ml) were prepared by dissolving in saline.

***Evaluating the relaxant effect***

According to a previous study, tracheal contractions were induced by methacholine and KCl for 5 min. Then cumulative concentrations of crocetin (0.02, 0.06, 0.14, and 0.3 mg/ml), petal extract of saffron (0.2, 0.4, 0.8, and 1 mg/ml), and theophylline (0.2, 0.4, 0.6, and 0.8 mM) or 1 ml normal saline (NS) were added to organ bath every 5 min and the effect of each concentration was evaluated before adding the next concentration. Then the concentration-response curve in each experiment was performed and the concentration of crocetin inducing 50% of the maximum relaxation effect (EC_50_) was calculated. In the incubated groups, TSM was incubated 10 min before and during the addition of various concentrations of crocetin (21).

***Data analysis***

The results were analyzed using a one-way analysis of variance (ANOVA) followed by Tukey’s multiple comparisons test. Data were expressed as mean ± SEM. InStat (GraphPad Software, Inc, La Jolla, USA) software was used, and *P*<0.05 was considered a significant level.

**Figure 1:**
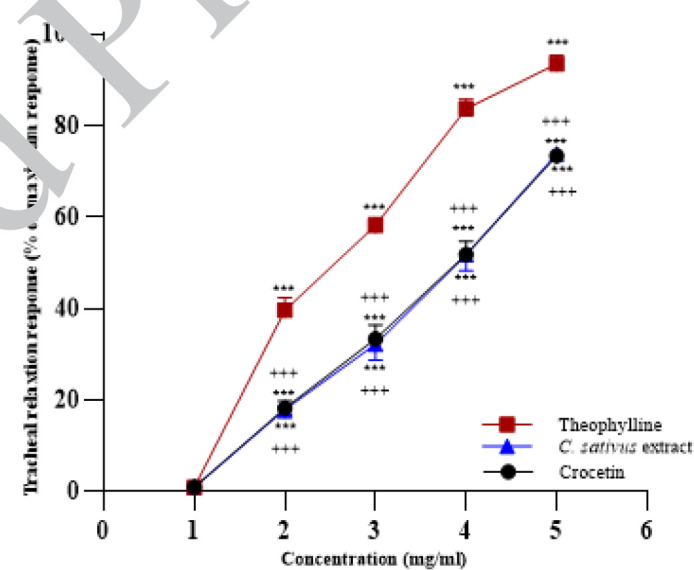
Concentration-response relaxant effects (mean ± SEM) of 0.02, 0.06, 0.14 and 0.3 mg/ml crocetin, 0.2, 0.4, 0.8 and 1 mg/ml petal extract of saffron and 0.2, 0.4, 0.6 and 0.8 mM theophylline tracheal smooth muscle (TSM) of rats contracted by 10 µM methacholine

Four concentrations of two agents were represented as 2, 3, 4, and 5 on the X-axis, *** *P*<0.001 compared to the effect of saline (1 on the X-axis). +++ *P*<0.001 compared to the effect of theophylline. ANOVA with Tukey Kramer post-test was used for statistical comparison (all, n =6).

**Figure 2:**
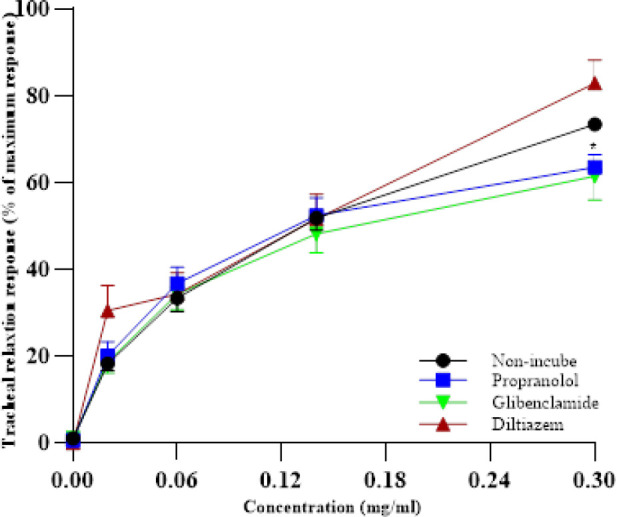
Concentration-response relaxant effects (mean ± SEM) of 0.02, 0.06, 0.14 and 0.3 mg/ml crocetin in non-incubated tracheal smooth muscle (TSM) of rats contracted by 10 µM methacholine (n=6), incubated TSM with diltiazem, propranolol and glibenclamide (n=7).

* *P*<0.05 compared to the effect of non-incubated TSM. ANOVA with Tukey Kramer post-test was used for statistical comparison.

**Figure 3:**
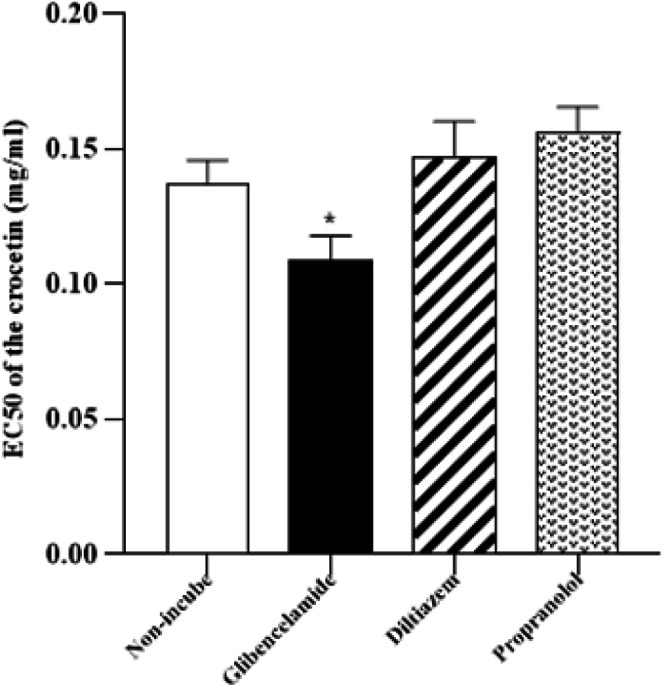
Level of 50% of the maximum relaxation effect (EC_50_) of crocetin in non-incubated tracheal smooth muscle (TSM) contracted by 10 µM methacholine, incubated TSM with glibenclamide, diltiazem, and propranolol

* *P*<0.05 compared to the effect of non-incubated TSM. ANOVA with Tukey Kramer post-test was used for statistical comparison.

**Figure 4:**
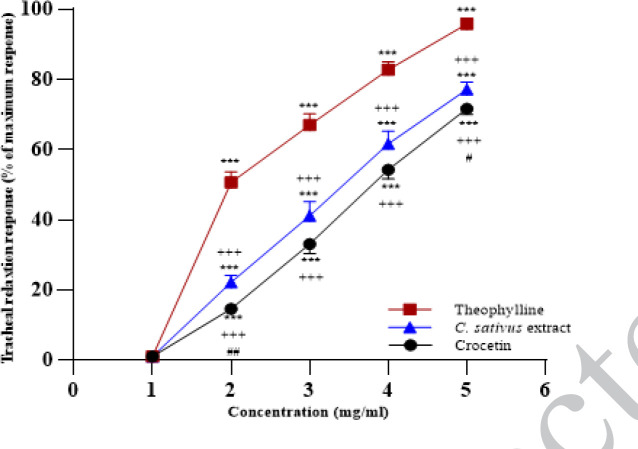
Concentration-response relaxant effects (mean ± SEM) of 0.02, 0.06, 0.14, and 0.3 mg/ml crocetin, 0.2, 0.4, 0.8, and 1 mg/ml petal extract of saffron and 0.2, 0.4, 0.6, and 0.8 mM theophylline in tracheal smooth muscle (TSM) contracted by 60 µM KCl. Four concentrations of two agents were represented as 2, 3, 4, and 5 in the X-axis

***P<0.001 compared to the effect of saline (1 in the X-axis). +++ *P*<0.001 compared to the effect of theophylline. # *P*<0.05 and ##*P*<0.01 compared to the effect of the extract. ANOVA with Tukey Kramer post-test was used for statistical comparison (all, n =6).

**Figure 5:**
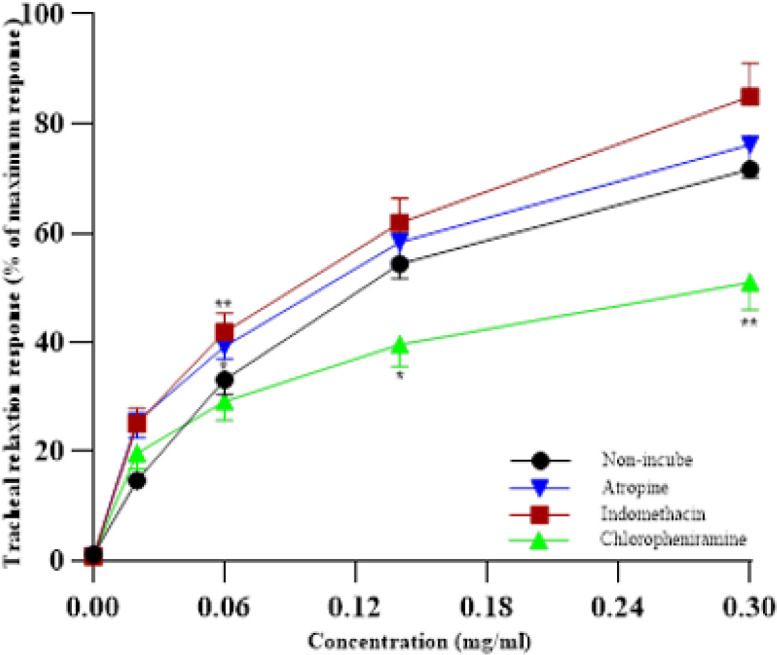
Concentration-response relaxant effects (mean ± SEM) of 0.02, 0.06, 0.14, and 0.3 mg/ml crocetin in non-incubated tracheal smooth muscle (TSM) contracted by 60 µM KCl (n=6), incubated TSM with indomethacin, atropine, and chlorpheniramine (n=7)

* *P*<0.05 and ** *P*<0.01 compared to the effect of non-incubated TSM. ANOVA with Tukey Kramer post-test was used for statistical comparison.

**Figure 6:**
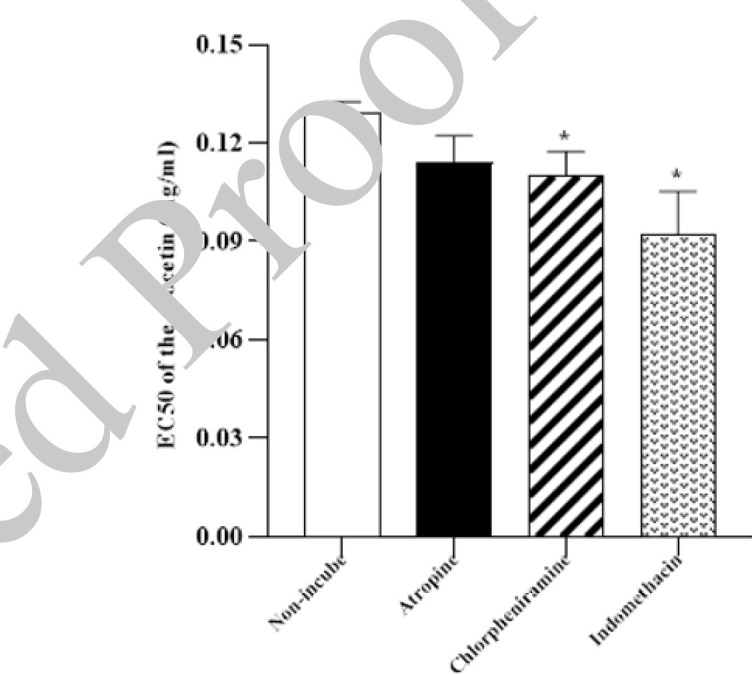
Level of 50% of the maximum relaxation effect (EC_50_) of the crocetin in non-incubated tracheal smooth muscle (TSM) contracted by 60 µM KCl, incubated TSM with atropine, indomethacin, and chlorpheniramine

*P<0.05 compared to the effect of non-incubated TSM. ANOVA with Tukey Kramer post-test was used for statistical comparison.

## Results

***Relaxant*** ***eﬀect on*** ***methacholine-induced contraction of TSM***

In the non-incubated TSM and contracted with methacholine, four concentrations of crocetin, the extract and theophylline showed concentration-dependent and marked relaxant eﬀect (all, *P*<0.001, Figure 1). The effects of different concentrations of crocetin and the extract were markedly lower than theophylline in the non-incubated TSM (all, *P*<0.001, Figure 1). There was no significant difference between the effects of crocetin and the extract.

In the incubated TSM with diltiazem (5 µM), the relaxation effect of the last concentration of crocetin was non-markedly higher than non-incubated TSM (Figure 2). The relaxation effect of the last concentration of crocetin in the TSM incubated with propranolol was lower than in the non-incubated TSM (*P*<0.05, Figure 2).

The level of EC_50_ crocetin in the incubated TSM with glibenclamide was markedly lower than in the non-incubated TSM (*P*<0.05, Figure 3). The levels of EC_50_ crocetin in the incubated TSM with diltiazem and propranolol were non-markedly higher than in the non-incubated condition (Figure 3).

***Relaxant*** ***eﬀect on KCl-induced contraction of TSM***

All concentrations of crocetin, the extract, and theophylline showed concentration-dependent and marked relaxant eﬀect on KCl-induced contraction of TSM in the non-incubated condition (all, *P*<0.001, Figure 4). In the non-incubated TSM, the relaxant effects of all concentrations of crocetin and the extract were markedly lower than those of theophylline (all, *P*<0.001, Figure 4). The relaxant effects of all concentrations of crocetin were significantly lower than those of the extract which were statistically markedly for the last and the first concentrations (*P*<0.05 and *P*<0.01, respectively, Figure 4).

The relaxant effects of the two last concentrations of crocetin in the incubated TSM with chlorpheniramine were markedly lower than the non-incubate TSM (*P*<0.05 and *P*<0.01 for the two last concentrations of crocetin, Figure 5). The levels of EC_50_ in the incubated TSM with chlorpheniramine and indomethacin were markedly lower than in non-incubated TSM (both *P*<0.05, Figure 6).

## Discussion

The relaxant effects of crocetin in pre-contracted TSM by methacholine and KCl were examined. To study the contributions of the possible mechanisms on the relaxant effects of crocetin, their concentration-response relaxant effects were examined in the presence and absence of competitive antagonists of each receptor, channel, or pathway and parallel right-ward shift and achieving a maximum response in the presence of each antagonist assessed.

In methacholine-contracted TSM, crocetin and the extract caused significant and concentration-dependent relaxant responses, but the effects of all its concentrations were significantly less than those of theophylline. Pharmacological agents such as propranolol, gilbenclamide, and diltiazem were used to investigate the possible mechanism of the relaxant effects of crocetin in methacholine-induced contraction of TSM.

The relaxant effect of crocetin in incubated TSM with glibenclamide was studied to assess the role of potassium channel opening property in its relaxant effect. Medium concentrations of crocetin showed a markedly lower relaxant effect in incubated tissue with glibenclamide than in non-incubated TSM. These findings suggested the contribution of the opening effect of potassium channels in the relaxant effect of crocetin on TSM. Different types of potassium channels in the airway with their determining role in the membrane resting potential as well as the effect on the release of neurotransmitters can regulate the response of the airways to contractile or relaxing agents (22).

The relaxant effect of two final concentrations of glibenclamide was non-significant and the EC_50_ value of crocetin was significantly lower than that of the non-incubated group, which probably indicates the effect of crocetin as a potassium channel opener. 

Stimulation of ß2-adrenergic receptors is the most probable mechanism for the relaxant effect of the drug on TSM. Therefore, to evaluate the role of ß2-adrenoceptors in the relaxant effect of crocetin, its effect on TSM incubated with propranolol and contracted with methacholine was examined. The relaxant effect of the last concentration of crocetin in the incubated TSM with propranolol was lower than in non-incubated tissue, and the EC_50_ value of crocetin in incubated tissue was higher than in non-incubated TSM. This finding indicates the stimulatory effect of crocetin on ß2- adrenoceptors as a proposed mechanism for its relaxant effect on TSM. In the study of Saadat *l*., crocin induced its relaxing effects by opening potassium channels and stimulating beta-adrenergic receptors which support the same mechanisms for the relaxant effect of crocetin in TSM (23).

In this study, the relaxant effects and EC_50_ value of crocetin were not different between non-incubated and incubated TSM with calcium channel inhibitor, diltiazem, which shows that this channel does not contribute to the relaxant effect of crocetin. Previous studies have suggested the role of crocetin on calcium channels, for example**,** the result of an experimental study shows that crocetin has an inhibitory effect on isolated cardiac muscle through its inhibitory effect on L-type calcium channels (24). 

Significant and concentration-dependent relaxant effects of crocetin in the non-incubated group contracted by KCl were observed. The relaxant effect of four concentrations of crocetin was markedly lower than that of theophylline. These findings indicated a relatively potent relaxant effect of crocetin. To investigate the role of muscarinic and histamine receptors as well as cyclooxygenase pathway in the relaxing effects of crocetin on TSM, its relaxant effects were examined on KCl-induced contraction of TSM in tissues incubated with atropine, chlorpheniramine, and indomethacin. 

The findings showed lower relaxant effects of crocetin in the TSM incubated with chlorpheniramine. Therefore, the inhibition of the histamine (H1) receptor is considered one of the mechanisms of the relaxant effect of crocetin in this study. Histamine as a chemical mediator plays an essential role in the occurrence of specific symptoms of asthma patients and the results of studies show that the level of histamine in the lung tissue of these patients is significantly higher than that of healthy people (25, 26). Since histamine induces bronchoconstriction of smooth muscle through its effect on type 1 histamine (H1) receptors, the clinical use of H1 antagonists has been common in the clinic for a long time (27).

Previous studies have mentioned similar mechanisms for the relaxing effects of saffron and its active ingredient on airway smooth muscle which is in line with the findings of the current study. Our previous studies showed histaminic antagonistic activity as a possible mechanism of the relaxing effects of saffron and safranal on TSM (28-30).

In the current study, the relaxant effect of crocetin was examined in TSM incubated with indomethacin to investigate the role of prostacyclins in the crocetin relaxant mechanism. The evidence shows the effect of cyclooxygenase (COX) expressed in the airway as a possible mechanism of AHR so that inhibition of cyclooxygenase caused bronchoconstriction in an experimental study (31, 32). In addition, clinical findings show that inhibiting COX in a group of patients with asthma can cause a condition called “aspirin-sensitive asthma”(33).

Statistical comparison of the relaxant effects of crocetin between non-incubated and indomethacin-incubated groups does not show any significant difference. However, the EC_50_ value of crocetin in incubated tissue with indomethacin was significantly lower than in non-incubated tissue. Therefore, the cyclooxygenase-dependent pathway is not involved in the relaxant effects of crocetin and the reason for the lower EC_50_ value of crocetin observed in incubated TSM with indomethacin is unclear to us.

The relaxant effects of crocetin in the group incubated with atropine were not significantly different compared to the non-incubated group and there was no significant difference in EC_50_ crocetin between the two groups. This finding indicated that the muscarinic receptor inhibitory effect did not contribute to the relaxant effect of crocetin. Considering the important role of muscarinic receptors in the physiopathology of asthma (34) previous studies have attributed the relaxing effects of saffron and its active ingredients to their inhibitory effect on these receptors (23, 35).

Although the relaxant effects of crocetin on some types of smooth muscles have been shown in previous studies, in the current study, its relaxant effects on smooth muscles of the airways and its possible mechanisms were shown for the first time. In spontaneously hypertensive rats, crocetin induced vasodilation via the endothelial nitric oxide (NO) pathway. Also, Llorens *l*. suggested that crocetin can promote endothelium-dependent relaxation on aortic contractility in rats with genetic hypertension (19). The results of another study showed that crocetin administration elevated urinary nitroxide metabolite (NO_2_/NO_3_) in hypertensive rats by reducing reactive oxygen species (ROS)-induced NO inactivation and decreased the systolic blood pressure in these animals. In hyper-cholesterolemic rabbits, the endothelium-dependent relaxant effect of crocetin on the thoracic aorta was due to increased production of nitric oxide (36). Thus, the results of the current study were supported by the mentioned previous studies.

The results of the present study indicated the potent relaxant effects of crocetin on TSM contracted by both methacholine and KCL, although its effects were significantly lower than the effect of theophylline at the concentrations used. The lower relaxant effects of crocetin compared to theophylline, may be caused by the low concentrations of these agents used in the present study or due to their low potency, which should be examined in future studies. These results indicated the bronchodilatory effects of crocetin. Therefore, the results suggested that crocetin could be used as a bronchodilatory agent in patients with obstructive pulmonary diseases. However, for this purpose, further clinical studies should be conducted in the future. 

## Conclusion

The results showed potent relaxation effects of crocetin on TSM although its effects were lower than theophylline at studied concentrations. The results also suggested that ß-adrenergic receptor stimulation, histamine (H_1_) receptor inhibition, and potassium channel opening contributed to the relaxant effect of crocetin on TSM.
